# Modeling the Population-Level Effects of Male Circumcision as an HIV-Preventive Measure: A Gendered Perspective

**DOI:** 10.1371/journal.pone.0028608

**Published:** 2011-12-20

**Authors:** Jonathan Dushoff, Audrey Patocs, Chyun-Fung Shi

**Affiliations:** Department of Biology and Institute of Infectious Disease Research, McMaster University, Hamilton, Ontario, Canada; INSERM and Universite Pierre et Marie Curie, France

## Abstract

**Background:**

Evidence from biological, epidemiological, and controlled intervention studies has demonstrated that male circumcision (MC) protects males from HIV infection, and MC is now advocated as a public-health intervention against HIV. MC provides *direct* protection only to men, but is expected to provide *indirect* protection to women at risk of acquiring HIV from heterosexual transmission. How such indirect protection interacts with the possibility that MC campaigns will lead to behavior changes, however, is not yet well understood. Our objective here is to investigate the link between *individual-level* effects of MC campaigns and long-term *population-level* outcomes resulting from disease dynamics, looking at both genders separately, over a broad range of parameters.

**Methods and Findings:**

We use simple mathematical models of heterosexual transmission to investigate the potential effects of a circumcision scale-up, combined with possible associated behavioral disinhibition. We examine patterns in expected long-term prevalence using a simple equilibrium model based on transmission factors, and validate our results with ODE-based simulations, focusing on the link between effects on females and those on males.We find that the long-term population-level effects on females and males are not strongly linked: there are many possible ways in which an intervention which reduces prevalence in males might nonetheless increase prevalence in females.

**Conclusions:**

Since an intervention that reduces long-term male prevalence could nonetheless increase long-term female prevalence, MC campaigns should explicitly consider both the short-term and long-term effects of MC interventions on females. Our findings strongly underline the importance of pairing MC programs with education, support programs and HIV testing and counseling, together with other prevention measures.

## Introduction

The HIV epidemic continues to exact a high toll, with 2.6 million new infections, and 1.8 million deaths, in 2009 [1]. Despite being home to only 10% of the world's population, Sub-Saharan Africa (SSA) represents two thirds of all persons living with HIV/AIDS, most of whom do not have access to anti-retroviral treatments [1]. Research into preventative vaccines, pre-exposure prophylaxis [2] and topical microbicides has yet to deliver an efficacious tool for preventing HIV transmission [3], [4]. Current efforts to halt HIV transmission are focused largely around education and voluntary counseling and testing (VCT), antiretroviral prophylaxis to prevent mother-to-child transmission, treatment for prevention [5], and recently, male circumcision (MC) campaigns [6], [7].

Evidence that MC provides partial protection to men against acquiring HIV infection from women has been accumulating for over 20 years [6]. Following randomized controlled trials (RCTs) in South Africa [8], Kenya [9], and Uganda [10] that demonstrated clinical efficacy of MC in preventing HIV transmission, the World Health Organization (WHO) recommended that promoting male circumcision be adopted as an anti-HIV strategy, while adding caveats about providing information, considering women's perspectives, and communicating fully and clearly, among others [7]. While MC holds promise to boost existing anti-HIV strategies, it also poses a complex set of issues because it provides only limited protection. In particular, it is important to guard against the danger of disinhibition – ie., that circumcised men will engage in riskier behavior, or that women will be more willing to engage in risky behavior with circumcised men [11].

Behavioural disinhibition is a pervasive feature of human behavior [12] and should be considered as a possible reaction to any protective intervention. Behavioural disinhibition has been documented in HIV microbicide trials [13], following anti-retroviral treatment rollout [14], and following negative HIV tests [15].There are as yet no studies of behavioral disinhibition in the context of population-scale MC interventions. Analysis of national population-based surveys in SSA showed that circumcised men are more likely to have concurrent partners than uncircumcised males [16], but these are ecological data, and do not specifically focus on interventions. A prospective, case-control study in Kenya compared men who chose to be circumcised with matched controls, and found no differences in risk behaviors at one year post-circumcision [17]. Although some indications of behavioral disinhibition were seen in the MC-intervention RCTs [8]–[10], these were not strong (and mostly not statistically significant). RCTs tend to focus on education, however, which can reduce risky behavior [18], and may therefore tend to suppress disinhibition.There is also the possibility that promotion of circumcision in mass circumcision campaigns will contribute to beliefs which increase disinhibition. Such beliefs are already present; for example a 2011 study of MC and disinhibition in three southern African countries found that a large proportion of respondents believed that circumcision was more protective than it is [19]. Thus, disinhibition should be considered a potential risk as MC campaigns go forward.

Studies of MC have mostly focused on effects on men. This is sensible, because MC directly protects men from infection, and – since women acquire HIV infection primarily from heterosexual transmission – direct protection for men translates to a certain amount of indirect protection for women. The possibility of behavioral disinhibition complicates this picture. Since there is no evidence for direct protection of women [20], [21], it is useful to ask whether an MC intervention that stimulates behavioral disinhibition could under some circumstances increase incidence in women while reducing incidence in men.

Women are disproportionately vulnerable to HIV infection in Africa, as evidenced by female prevalence rates that exceed male rates in nearly every country in SSA [22]. While this trend may be partially driven by biological factors, unbalanced gender-power relationships and violence against women also play important roles [23]. Many more male than female respondents in SSA population surveys report multiple sexual partners in the previous year, and many females do not believe they have the right to refuse sex to partners [24]. These social factors contribute to a situation where male beliefs and behaviors have a large influence on female HIV risk.

A wide range of mathematical models have predicted reductions in HIV prevalence following large-scale MC [25], including two that acknowledged the possibility that female prevalence could go up even as male prevalence goes down in the presence of behavioral disinhibition [26], [27]. We are not aware, however, of studies that systematically explore how closely linked we should expect effects on males and females to be.

Here, we use a simple equilibrium analysis, based on “transmission factors” [28], and taking individual-level heterogeneity in sexual mixing into account (using the approach of [29]). Transmission factors are analogous to the basic reproductive number, but instead ask how many cases will an average case *in a given gender* produce in a susceptible population *of the other gender*.The expected relationship between transmission factors and the predicted equilibrium prevalence in each gender is robust to assumptions about details of transmission and progression, including contact rates, probability of transmission and duration of latency and infectiousness.

Thus, the transmission factor approach allows us to explore the balance between protection and disinhibition in women and men over a wide range of parameters, and ask how broad are the parameters under which effects on men and effects on women move in different directions – ie., how safe is the assumption that an MC intervention with net benefits for men will also have net benefits for women?

Transmission factors are products of risk factors such as contact rates, duration of infectiousness and transmission probabilities. In this model, the effect of different mechanisms of protection and disinhibition on predicted equilibria depends only on how they affect the two transmission factors. We validate our conclusions from the equilibrium analysis using more detailed simulation models, which show very similar qualitative behavior for the selected parameters. For simplicity in addressing the question of how closely linked two genders are in a heterosexually transmitted disease, we do not consider homosexual transmission, nor mother-to-child transmission.

## Methods

### Equilibrium calculations

The transmission factor approach [28] allows equilibrium prevalence in each gender to be estimated based on unitless transmission factors reflecting overall risk of transmission from each gender to the other. We incorporate a phenomenological response to prevalence as a proxy for heterogeneity [29]. Thus, we solve two simultaneous equations of the form:
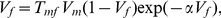
(1)to obtain 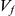 and 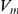, the proportion of sexually active females and males respectively “affected” by the disease if it reaches equilibrium. 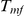 is the “transmission factor” describing transmission *from* males *to* females. The equilibrium equation for the male population is exactly symmetric.

The parameter 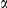 sets the strength of the heterogeneity response. If we set 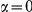, we would have a classic homogeneous model. In a heterogeneous population, we expect effective transmission to be reduced as prevalence increases, largely because the average contact rate and susceptibility of those in the susceptible pool decrease as the most susceptible individuals move to the infected class [29], [30]. To explore the effects of interventions and behavior changes on equilibrium prevalence, we hold the value of 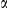 constant, and calculate equilibria over a wide range of values of the transmission factors, which incorporate the various risk behaviors that determine the average number of new potential infections that are generated by each infection. We obtained the value 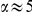 using crude (least-squares) fits to prevalence data from antenatal clinics in sub-Saharan Africa – we use it here as a reasonable example, and not to suggest we have described the heterogeneity response in detail.

### Dynamical simulations

**Figure 1:**
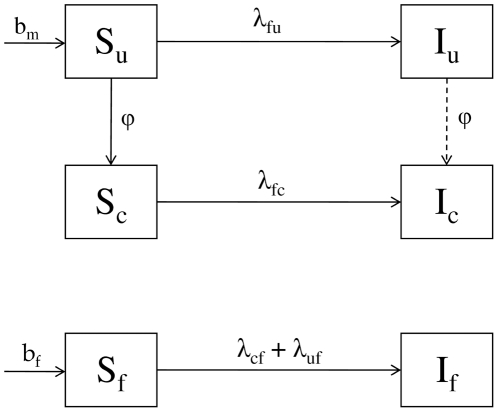
Simplified model diagram. Disease is transmitted among females, and circumcised and uncircumcised males (indexed by 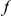, 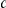 and 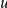. Susceptible individuals (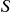) exposed to infection move to infectious classes 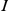). 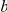 represents “births” (really, recruitment to sexual activity) in males and females respectively. 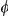 is the rate at which males become circumcised; it is calculated to give the desired equilibrium proportion of circumcised susceptible males. The forces of infection are given by 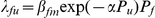, 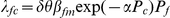, 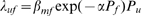, 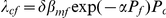, where 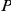 is prevalence in each class, 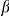
s are intrinsic transmission rates, and 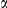 is the heterogeneity parameter. 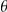 represents direct protection of circumcised males, and 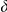 represents disinhibition. Our ODE model has four identical sub-boxes for each infectious box, to better match the time distribution of the infectious period [29].

Our dynamical model is diagrammed in Figure 1. We divide the sexually active population into females, uncircumcised males and circumcised males; each group can be either susceptible or infected. We further divide the infected classes into equivalent subclasses, to achieve a more realistic distribution of time to death [29]. We model this dynamical system using a standard ODE approach.

The model parameters are given in Table 1. They are broadly consistent with what is known about HIV transmission in southern Africa, and with other recent transmission models [29]–[31]. Males and females enter the susceptible (sexually active) population at a constant rate, and become infected at a rate proportional to the proportion of heterosexual partners infected and the transmission risk associated with a given sexual encounter.

**Table 1:** Parameters used in simulations shown here.

Parameter	Value	Source
HIV prevalence at MC implementation	25%	Assumption*
Proportion circumcised at baseline	15%	[38]
Equilibrium proportion circumcised after intervention	80%	Assumption*
Average time spent in susceptible class	40 yrs	[39]
Reproductive number 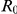	5	[30], [37]
Heterogeneity factor 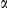	5	Unpublished fit, *see text*
Average time spent in infectious class	10 yrs	[40]
Direct protection for circumcised males	60%	[8]–[10]
Relative transmission rate 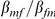	2	[32], [34]
Behavioral disinhibition factor	1.5	Assumption*

*Assumptions chosen for illustrative purposes in dynamical simulations.

Estimates of the difference in transmission direction (i.e. male-to-female versus female-to-male) suggest males are very roughly twice as likely to transmit HIV to female partners as females are to males, but these estimates cover a broad range [32]–[34]. The results shown here assume a 2∶1 ratio; results with a 1∶1 ratio are qualitatively similar.

Transmission to males in our model is reduced a further 60% if the male is circumcised [8]–[10]. We assume that females receive no direct protection from circumcision of male partners [20], [21]. We do not consider the short-term effect of interruption of sexual activity due to the circumcision procedure.

Baseline simulations qualitatively match observed patterns for southern African countries (see [29]). In the intervention scenarios,the rate 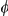 at which individuals are circumcised increases in the year 2010 (Figure 1). We increase 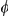 to a level which leads the prevalence of MC to increase gradually from 15% to 80%. In disinhibition scenarios, the total contact rate (representing risk behaviors) increases either for all circumcised men, or only for men who were HIV- when circumcised (see Discussion).

### Parameters

**Figure 2:**
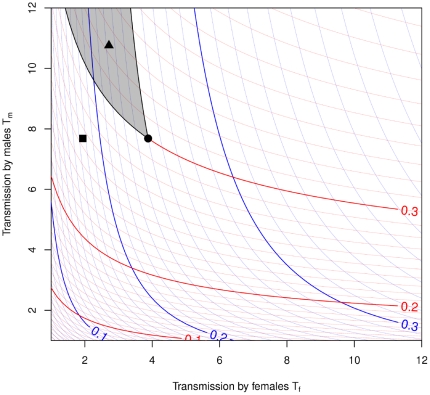
Expected population-level equilibrium prevalence as a function of individual-level “transmission factors”. Contours for women's (men's) prevalence are shown in red (blue). The shapes show the pre-intervention equilibrium (solid circle), and possible long-term effects of an intervention which results in direct protection for men without (square) and with (triangle) an increase in risky behavior due to disinhibition. The heterogeneity parameter is 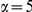. The shaded area shows the region in which equilibrium prevalence in women increases while that in men decreases.Other combinations of parameters, and parameter changes,, and parameter changes, can be evaluated in a similar fashion, by comparison with the red and blue lines.

It is worth noting that our equilibrium curves (Figure 2) depend on only one parameter: the heterogeneity response 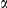. Interpretation of their meaning depends additionally on five other parameters: the transmission factors 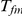 and 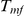, the direct protection afforded to each gender by circumcision (here we assume females are not*directly* protected), and an assumption about disinhibition.

A list of parameters for the simulation model is given in Table 1. Model parameters are derived from literature and public data, and are consistent with those used in similar models based in SSA [26], [27], [30], [31], [35]. The simulation model is intended primarily to validate (and illustrate) the broader conclusions of the equilibrium model.

### Code

All calculations were made using the free, open-source statistical package R [36]. Code for all calculations, and for producing all of the figures, is available at http://lalashan.mcmaster.ca/hivcirc/and can be used freely for non-commercial purposes.

## Results

The results of the equilibrium model are shown in Figure 2. This simple model predicts long-term equilibrium prevalence as a function only of the transmission functions and the heterogeneity parameter. Thus, disinhibition due to increased contact rate or reduced condom use is modeled by proportionally increasing both transmission factors, while protection of men by circumcision is reduced by decreasing transmission from females.

The red (blue) contours show expected equilibrium prevalence in women (men) as a function of the level of effective transmission *by* each gender. In other words, the figure shows long-term risk to each gender (measured as equilibrium prevalence) as a function of short-term risk factors (reflected in the transmission factors). Thus, for example, as we move to the right (corresponding to increased transmission by women to men), we move “up” (away from the origin) relative to both the blue and red contours. However, we pass rapidly through blue contours (indicating rapidly increasing long-term risk to men), but more slowly through red contours (indicating less rapidly increasing long-term risk to women).

This picture allows us to visually evaluate the extent to which long-term risk to women and long-term risk to men are linked in a simple heterosexual transmission model. Starting from any contour intersection, we can ask how much latitude there is for parameter changes that have a positive effect on one gender but a negative effect on the other. If the effects of change on the two genders were closely linked, the red curves and blue curves would be nearly parallel, and there would be very little room to move in a way that is good for one gender but bad for another. Here, however, the curves are not closely matched – a change that increases transmission by men while decreasing transmission by women (at the individual level) is likely to reduce long-term risk to men and increase long-term risk to women, despite the fact that the two genders are linked by heterosexual transmission.

As an example, the closed circle illustrates plausible parameters for a southern African country (and the long-term expected proportion ofthe sexually active population in each gender affected by HIV if these parameters don't change). The square shows how these values would change under a hypothetical circumcision intervention that directly reduces transmission to men (as a group) by 40%, corresponding loosely to increasing circumcision from 15% to 80% of the population, with a 60% protective effect for circumcised individuals. The open circle shows the effect of additionally increasing risky behavior in the whole population by 25% (corresponding to an assumed larger increase for interactions involving circumcised men). In this example, as for many other examples with similar parameters, the effect of circumcision alone is good for both genders in the long run, while the effect of circumcision with disinhibition is good for men but bad for women.

**Figure 3:**
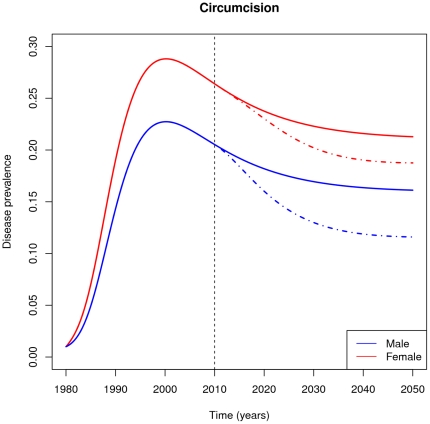
HIV prevalence response to a circumcision intervention. Curves show prevalence through time in females (red) and males (blue) in a hypothetical population with (dashed lines) and without (solid lines) the introduction of circumcision in year 2010. We assume that circumcision does not change behavior.

Figure 3 shows simulation results for HIV prevalence in men and women for a hypothetical population. Baseline curves approximate the rate of growth in the early epidemic and peak in the mid 1990's, as observed in some high-prevalence sub-Saharan African countries. The baseline scenario is modified in 2010, when the rate of circumcision is scaled up to a level that eventually achieves 80% equilibrium coverage, providing direct protection for men (60% protective effect).

**Figure 4:**
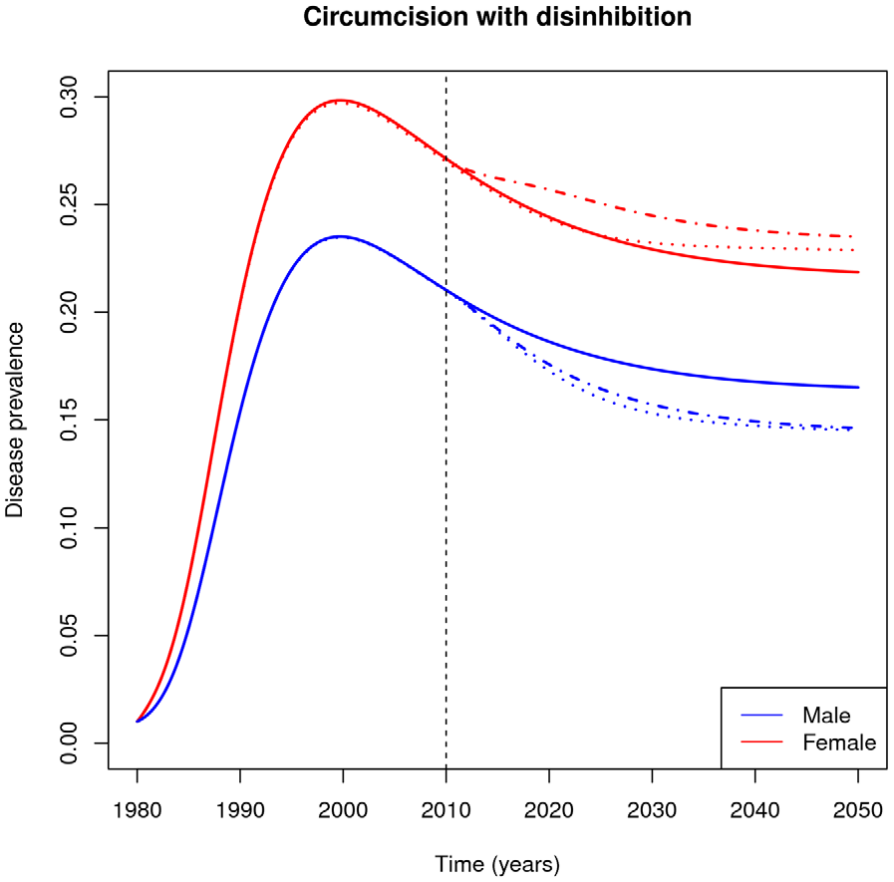
HIV prevalence response to a circumcision intervention with behavioral disinhibition. Curves show prevalence through time in females (red) and males (blue) in a hypothetical population with (dashed lines) and without (solid lines) the introduction of circumcision in year 2010. We assume that behavioral disinhibition leads to a 50% increase in effective transmission for interactions involving circumcised males. Dotted lines show the case where disinhibition does not occur among individuals who were HIV-positive at the time of circumcision (see text).

Figure 4 shows the same population, but with a 50% increase in effective transmission for interactions involving circumcised males (dashed line). Here, males as a group continue to benefit from the intervention, but female prevalence increases, relative to baseline. The dotted line shows the same simulation under the assumption that increased risk behavior does not occur in men who are HIV+ before circumcision (this could happen if they are tested and counselled at the time of circumcision, or if they do not get circumcised). In the short-term, avoiding disinhibition among such men greatly ameliorates negative effects on women (the dotted line diverges before the intervention, because we assume men who are circumcised for any reason behave differently in these scenarios). In the longer term, the two scenarios become similar, since fewer and fewer men will be infected before circumcision after many years of an active campaign.

## Discussion

We have used a simple, robust equilibrium model to show that even in a population where HIV transmission is predominantly heterosexual, the link between effects on male prevalence and effects on female prevalence is only moderately strong. Simple simulations confirm these results, and underline the possibility that interventions that benefit one gender (on average) may prove harmful to the other in some cases.

The key to this somewhat counter-intuitive scenario is the possibility that, in the presence of behavioral disinhibition, fewer infectious men could infect a large number of women, who are not directly protected by circumcision. Despite the risk posed by both increased risky behavior and increased prevalence in women, however, the prevalence in men could still remain low because of the large protective effect of circumcision.

Even if the net effect of MC is to reduce overall prevalence, an increase in prevalence among females would be of concern. The gender ratio of HIV cases itself has an effect on how disease is perceived. In a region like SSA, where women have a higher HIV prevalence rate and face unique barriers to prevention, treatment, and social support following HIV/AIDS diagnosis partly because of gender inequity [23], MC campaigns must incorporate a gendered perspective.

Behavioral disinhibition is a common response to effective interventions. The success of MC in the long term will depend on the ability of public-health workers to control *messages* – for example, whether people believe that they need to practice safe sex after circumcision, or that circumcised men are always HIV-negative. MC is irreversible and may have long-term impacts on behaviors; it is crucial that MC campaigns are careful about the messages that they communicate.

Our simulation results suggest potential negative consequences of circumcising HIV-positive men (given the subsequent disinhibition of these men or their partners), with disproportionate costs to women. On the other hand, there are potential drawbacks to attempting to exclude HIV-positive men from circumcision programs, including confidentiality, the possibility of stigma, and the possibility that such men would receive medical benefits (e.g., reduction in other STIs) from such a procedure.

Our simulation results show a relatively small overall effect of circumcision rollout. This is a direct result of our incorporation of strong, individual-level heterogeneity. Our approach to both modeling population heterogeneity and estimating its magnitude is crude, and more detailed methods [26], [37] may provide better estimates of population-level outcomes. We believe that the effects of heterogeneity are substantial, but our main qualitative finding – that male and female outcomes are not tightly linked – is not at all sensitive to our heterogeneity assumptions, or indeed to the presence of heterogeneity.

The possibility that behavioral disinhibition may accompany male circumcision does not argue against the introduction of an intervention that has the potential to reduce HIV transmission. Our results do, however, underline the need for a better understanding of the behavior implications of MC campaign and for an explicitly gendered perspective during clinical trials, intervention planning and program evaluation.
